# Evolution in diagnosis and management of chronic liver diseases

**DOI:** 10.1002/ueg2.12502

**Published:** 2023-11-28

**Authors:** Christophe Moreno, Xiaolong Qi

**Affiliations:** ^1^ Department of Gastroenterology, Hepatopancreatology and Digestive Oncology Hôpital universitaire de Bruxelles Université Libre de Bruxelles Brussels Belgium; ^2^ Center of portal hypertension, Department of radiology Zhongda Hospital, School of Medicine, Southeast University Nanjing China

**Keywords:** chronic liver disease, complications of cirrhosis, diagnosis, hepatocellular carcinoma, severity assessment, steatotic liver disease, therapeutic management

Over the past decade, the epidemiology of chronic liver disease has evolved considerably, with a decrease in the prevalence of chronic hepatitis C thanks to major therapeutic advances, and a growing increase in steatotic diseases associated with metabolic syndrome. Significant progress has also been made in understanding the pathophysiology, natural history, prognosis and therapeutic management of chronic liver diseases and its complications. To summarize and highlight the latest evidence and make it available to healthcare professionals, we, hereby, present a “Liver special issue” focused on the most recent advances in diagnosis, prognosis and therapeutic management of chronic liver diseases and its complications.

## STEATOTIC LIVER DISEASE: A GROWING CHALLENGE FOR THE FUTURE

Steatotic liver diseases, mainly Nonalcoholic fatty liver disease (NAFLD) and alcohol‐related liver disease (ALD), are the leading causes of chronic liver disease worldwide. Although NAFLD and ALD share many pathophysiological, histological and genetic features, natural history of these two diseases is dramatically different, ALD being associated with a higher risk of cirrhosis and liver‐related events. However, in clinical practice, alcohol consumption and features of metabolic syndrome often coexist. Hagström H et al highlighted the scientific evidence suggesting an interactive effect between alcohol consumption and metabolic risk factors (in particular type 2 diabetes and obesity) that are associated with the risk of progressive fibrosis and development of cirrhosis.^1^

Type 2 diabetes has emerged as the most important risk factor associated not only with the development of NAFLD, but also for the progression of liver fibrosis and the occurrence of liver‐related complications. In their review,^2^ Otero‐Sanchez L et al described the complex interplay between type 2 diabetes and liver‐related outcomes, in particular the increased risk of liver decompensation and Hepatocellular carcinoma (HCC) in patients with NAFLD and diabetes. This strongly supports the recommendations endorsed by the American association of clinical endocrinology, American association for the study of liver diseases (AASLD), the European association for the study of the liver (EASL), and the European association for the study of diabetes to screen population with type 2 diabetes for liver fibrosis. Given the high prevalence of NAFLD patients worldwide, it is essential to identify those at risk of progressing to cirrhosis and its complications in a simple, non‐invasive way. Although liver biopsy remains the gold standard for diagnosing non alcoholic steatohepatitis (NASH) and staging liver fibrosis, a number of non‐invasive tests for assessing liver fibrosis have been developed and demonstrated to be effective in detecting advanced fibrosis and liver‐related outcomes. Li J and colleagues nicely and critically review the different tools to diagnose NAFLD and assess disease severity.^3^ What is still lacking are strong data to support the use of non‐invasive tests to monitor the effect of therapeutic interventions in NAFLD patients. Lifestyle modification is the cornerstone of management in patients with NAFLD. Several NASH‐specific drugs are in advanced clinical development and will enrich our therapeutic options in NASH‐patients with significant fibrosis before the cirrhotic stage, in a near future.

Recently, a Delphi consensus panel proposed a new nomenclature and diagnostic criteria for fatty liver disease, which were endorsed by EASL and AASLD.^4^ The main aim of this new nomenclature was to avoid the stigmatizing terms “alcoholic” and “fatty”. Excessive fat accumulation in the liver is now defined as “steatotic liver disease” (SLD). According to this new nomenclature, NAFLD is now termed Metabolic‐Associated Steatotic Liver Disease. This nomenclature also introduced a group in which metabolic and alcohol risk factors coexist, termed MetALD. Although patients with MetALD are frequently seen in clinical practice, this term raises a lot of questions in the scientific community. Among them, we have to consider that risk of liver disease progression and liver‐related complications are drastically higher in patients with ALD.

Alcohol‐related liver disease represents indeed the most common indication for liver transplantation (LT) in Western countries. Outcomes of LT for ALD are excellent and comparable with those of LT for other aetiologies. An important debate in recent years has concerned the period of abstinence required before transplantation, particularly for patients with severe alcoholic hepatitis who have failed to respond to medical treatment, for whom early LT has been shown to be a highly effective therapeutic option with an acceptable risk of relapse in appropriately selected patients. In their review,^5^ Germani G et al describe the necessary pre‐transplant evaluation, as well as the monitoring to be carried out after transplantation in order to obtain the best long‐term results.

## VIRAL HEPATITIS: THE LEAST PREVALENT IS NOT FORGOTTEN

For a long time, hepatitis Delta (HDV) was the forgotten virus. However, HDV is by far the most aggressive form of viral hepatitis. Progression of liver disease is more rapid for those with HDV compared to those mono‐infected by hepatitis B virus (HBV) or hepatitis C virus. The risk of cirrhosis and HCC is significantly higher than with chronic hepatitis B alone. Epidemiology of HDV is uncertain because data are entirely absent for many countries, and because only a small proportion of HBsAg‐positive individuals are tested for HDV. Recent data suggest that the best way to improve HDV screening is to perform reflex testing of anti‐HDV antibodies in the new HBsAg positive cases. A recent 2023 Clinical Practice Guidelines on HDV Virus by EASL recommends that screening for anti‐HDV antibodies should be performed at least once in all HBsAg‐positive individuals.^6^ Until recently, the only treatment option for individuals with chronic hepatitis D was interferon alfa given for at least 48 weeks. Response rates tend to be low and treatment is associated with considerable side‐effects. Bulevirtide—a synthetic lipopeptide that inhibits entry of HDV and HBV into hepatocytes—represents a new treatment option and has been recently licensed in the EU. In their article,^7^ Buti M et al reviewed epidemiology, natural history of HDV disease. They also summarized results with Bulevirtide either in monotherapy or in combination with PegIFN, as well as other therapeutic options under development. The expansion of treatment options probably offers an opportunity to embed HDV more firmly in elimination plans.

## COMPLICATIONS OF CIRRHOSIS: TO A BETTER PROGNOSIS AND THERAPEUTIC MANAGEMENT

Cirrhosis is prevalent worldwide and morbid. It evolves from a compensated stage to decompensated stage, the latter defined by the development of complications of ascites, variceal hemorrhage and hepatic encephalopathy. Survival is entirely different depending on the stage. The median survival time following onset of hepatic encephalopathy and ascites is 0.92 and 1.1 years, respectively. Among people with ascites, the annual incidence of hepatorenal syndrome is 8%, which is associated with a median survival of less than 2 weeks.^8^ Treatment with non‐selective beta‐blockers prevents decompensation in patients with clinically significant portal hypertension. In patients with acute variceal hemorrhage, transjugular intra‐hepatic porto‐systemic shunt (TIPS) improves mortality and has become the standard of care in many centers; and retrograde transvenous obliteration and/or variceal cyanoacrylate injection have emerged as alternatives to TIPS for patients with bleeding from gastrofundal varices.^9^ In patients with ascites, the standard care includes sodium restriction and combination loop and aldosterone antagonist diuretics.^8^, ^10^ Emerging evidence suggests that TIPS might be used earlier before strict criteria for refractory ascites are met, and long term albumin use is under assessment for patients with uncomplicated ascites.^9^ The first line therapy for hepatorenal syndrome is the combination of terlipressin and albumin.^9^ Current best‐practice for the management of hepatic encephalopathy includes nutritional support as well as therapeutic agents that mitigate the effects of factors in the causal pathway for the disease process. All patients with hepatic encephalopathy are recommended to consume 1 g/kg dietary protein and supplementary branch‐chain amino acids. Lactulose and rifaximin are first and second‐line treatment for hepatic encephalopathy.^9^, ^10^ However, there are still some controversial areas where the evidence base is less clear or opinions amongst practitioners remain divided, further confirmatory studies are needed beyond the current guidelines.

## HEPATOCELLULAR CARCINOMA: A START OF A NEW THERAPEUTIC ERA?

Hepatocellular carcinoma is one of the most common cancers and a leading cause of cancer‐related mortality. According to Barcelona Clinic Liver Cancer staging and treatment strategy, ablation and surgical treatment, transarterial chemoembolization (TACE) and systemic treatment are the standard of care for early, intermediate, and advanced stage HCC, respectively.^11^ In recent years, immunotherapies have revolutionized the treatment landscape of HCC. The combination of immune checkpoint inhibitors (ICIs) and molecular targeted agents (MTAs), such as atezolizumab and bevacizumab,^12^ and camrelizumab plus rivoceranib,^13^ have demonstrated superior survival advantages compared to sorafenib in advanced HCC patients. There is considerable rationale for integrating TACE, ICIs, and MTAs in HCC treatment. The CHANCE001 study suggested that TACE plus ICIs, and MTAs could signiﬁcantly improve progression‐free survival and overall survival relative to TACE monotherapy in real world practice.^14^ In the past, there are no established adjuvant treatment for patients with high‐risk recurrence HCC. Recently, IMbrave050 study reported that atezolizumab plus bevacizumab could improve recurrence‐free survival versus active surveillance in patients with high‐risk recurrence HCC following curative‐intent resection or ablation, markig “the first step towards adjuvant therapy in HCC”.^15^ However, the longer follow‐up is still needed to further evaluate both recurrence‐free and overall survival. There are still some ongoing phase 3 trials exploring immunotherapies alone or in combination across various stages of HCC, which may also reshape the treatment landscape.

In summary, this special issue covers a comprehensive review of the major advances in chronic liver disease management. The information herein provided will be instrumental in inspiring our healthcare professionals to improve diagnosis and treatment of patients with chronic liver disease.

## CONFLICT OF INTEREST STATEMENT

The authors have no conflicts of interest to declare.

## Data Availability

Data sharing is not applicable to this article as no new data were created or analyzed in this study.
